# Transcriptomic dataset for early inflorescence stages of oil palm in response to defoliation stress

**DOI:** 10.1016/j.dib.2022.107914

**Published:** 2022-02-03

**Authors:** Ardha Apriyanto, Walter Ajambang

**Affiliations:** a Research and Development, PT. Astra Agro Lestari Tbk, Jl. Puloayang Raya Blok OR I, Kawasan Industri Pulogadung, Jakarta Timur, Indonesia; b Biopolymer Analytics, Institute of Biochemistry and Biology, University of Potsdam, Karl-Liebknecht-Str. 24-25, Building 20, Potsdam-Golm, Germany; c Institute of Agricultural Research for Development, Oil Palm Research Centre. BP 243, Douala, Cameroon

**Keywords:** Complete defoliation, Flower development, Leaf axil, NGS, RNA-seq, Sex determination

## Abstract

Oil palm breeding and seed development have been hindered due to the male parent's incapacity to produce male inflorescence as a source of pollen under normal conditions. On the other hand, a young oil palm plantation has a low pollination rate due to a lack of male flowers. These are the common problem of sex ratio in the oil palm industry. Nevertheless, the regulation of sex ratio in oil palm plants is a complex mechanism and remains an open question until now. Researchers have previously used complete defoliation to induce male inflorescences, but the biological and molecular mechanisms underlying this morphological change have yet to be discovered. Here, we present an RNA-seq dataset from three early stages of an oil palm inflorescence under normal conditions and complete defoliation stress. This transcriptomic dataset is a valuable resource to improve our understanding of sex determination mechanisms in oil palm inflorescence.

## Specifications Table

**Table:** 

Subject	Biological sciences
Specific subject area	Omics: Transcriptomics
Type of data	Table Figure
How the data were acquired	Paired-end sequencing on Illumina HiSeq 2000 platform
Data format	Raw - Fastq Analyzed - Count Table
Description of data collection	Total RNA was extracted from three different inflorescence stages of control (untreated) and defoliation stress (treated)
Data source location	Institution: Research and Development, PT. Astra Agro Lestari Tbk City/Town/Region: Pangkalan Lada, Kalimantan Tengah Country: Indonesia Latitude and longitude for collected samples/data: (2°25′36.0′′ S, 111°47′08.1′′ E).
Data accessibility	All data in this article are available at NCBI, BioProject (PRJNA769249) Direct URL to data: https://www.ncbi.nlm.nih.gov/bioproject/769249 and Gene Expression Omnibus (GSE186394) Direct URL to data: https://www.ncbi.nlm.nih.gov/geo/query/acc.cgi?acc=GSE186394
Related research article	*W. Ajambang, S.W. Ardie, H. Volkaert, G.F. Ngando-Ebongue, S. Sudarsono, Comparative expression profiling of three early inflorescence stages of oil palm indicates that vegetative to reproductive phase transition of meristem is regulated by sugar balance, Functional plant biology FPB 42 (2015) 589–598.*https://doi.org/10.1071/FP14343*.*

## Value of the Data

• This transcriptomic dataset is a valuable resource for investigating the biological mechanism of inflorescence development in oil palm.
• The data provide transcriptomic alterations caused by defoliation stress of early inflorescence stages of oil palm.
• The dataset can be used to accelerate research on elucidating the mechanism of oil palm sex determination.

## 1 Data Description

**Fig. 1:**
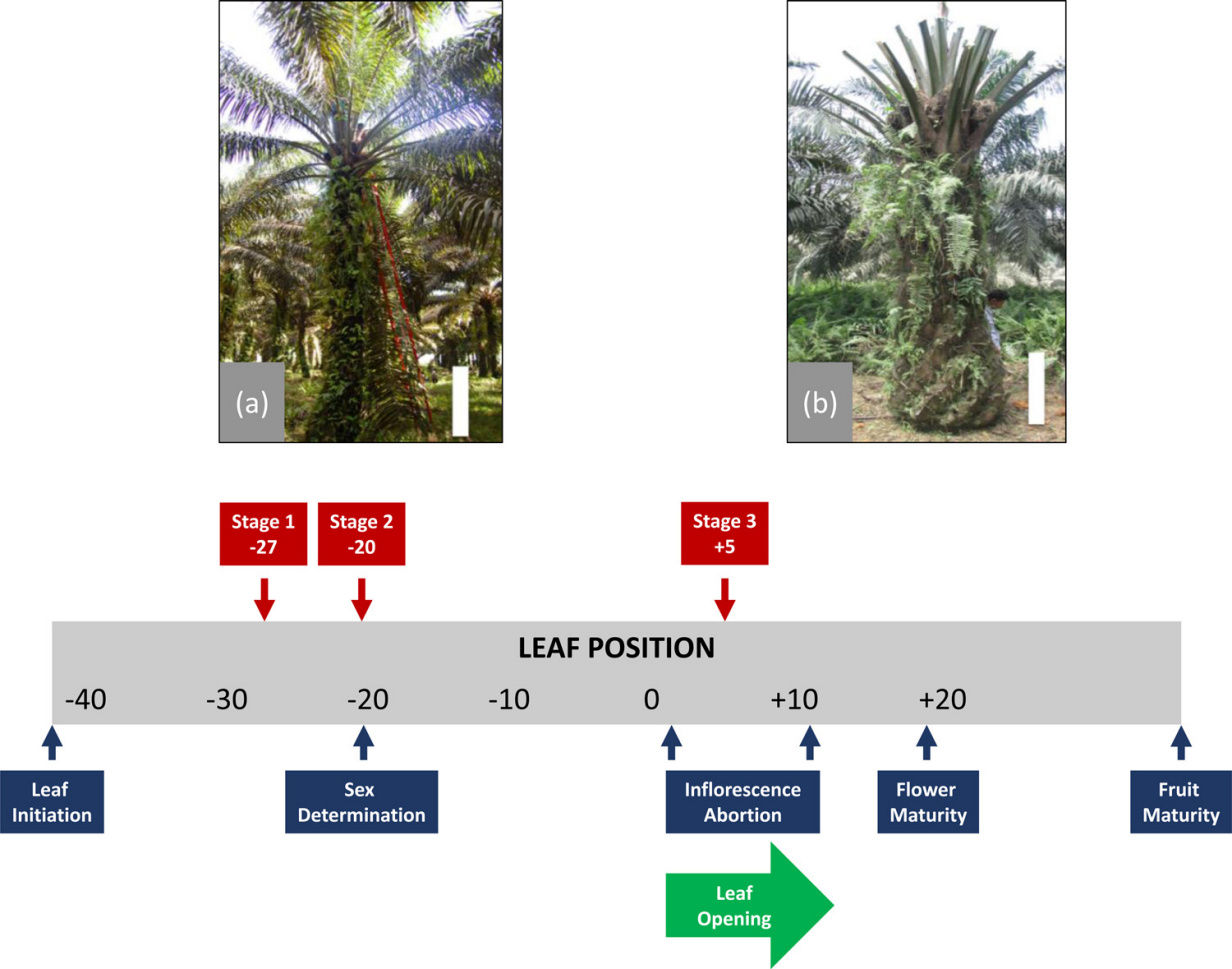
The oil palm plant in the field and the design experiment for obtaining this dataset. Plant with normal condition (a) and complete defoliation (b). Bar scale = 1 m. The schematic of inflorescence development was adapted from [2].

The transcriptome dataset of three early phases of an oil palm (*Elaeis guineensis* Jacq.) inflorescence under normal conditions and complete defoliation stress is described in this article. The design experiment and plant used for the data collection can be seen in Fig. 1. and the detail about the research background can be seen in a related research article [1].

Transcriptomic data for each sample of early inflorescence stages were obtained by sequencing using an Illumina HiSeq 2000 platform. All sequencing data were deposited in NCBI Sequence Read Archive (SRX12511660-SRX12511665) under a bioproject accession number PRJNA769249 shown in Table 1 and Gene Expression Omnibus (GEO) under accession number GSE186394.

**Table 1:** Transcriptomic dataset of early inflorescence stages.

No	Developmental Stage	Treatment	SRA	Reads	Mapped Reads (%)
1	Inflorescense at leaf axil -27	control	SRX12511664	7.046.558	79.32
2	Inflorescense at leaf axil -20	control	SRX12511663	7.129.648	78.83
3	Inflorescense at leaf axil +5	control	SRX12511662	6.697.791	79.30
4	Inflorescense at leaf axil -27	stress	SRX12511661	8.337.904	78.96
5	Inflorescense at leaf axil -20	stress	SRX12511660	7.256.915	78.40
6	Inflorescense at leaf axil +5	stress	SRX12511665	7.923.290	79.78

**Fig 2:**
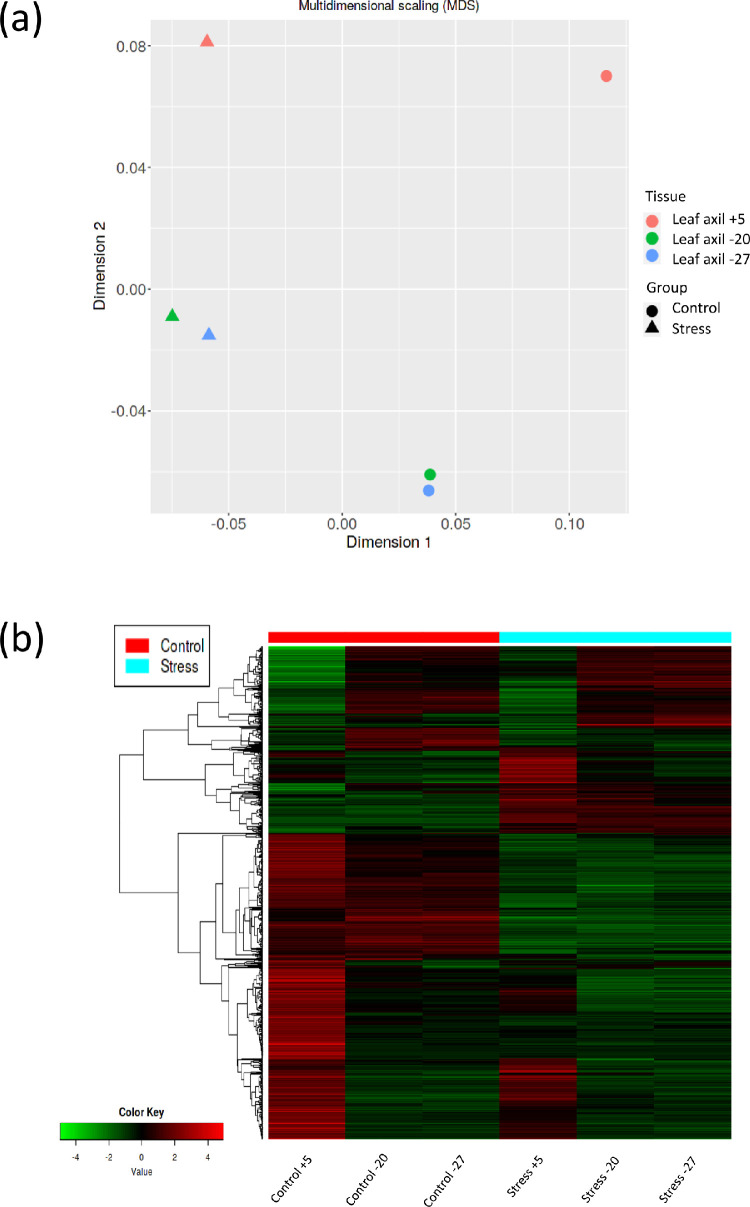
Clustering analysis among samples using multidimensional scaling (a) and heatmap (b).

In total, 44.392.106 reads were reported with 78.4 - 79.32% mapped reads, which indicates a good mapping result to oil palm genome reference (GCF_000442705.1). The calculated Fragments Per Kilobase Million (FPKM) values for differential expression profiling analysis can be seen in Table S1 (10.6084/m9.figshare.19091951). Multidimensional scaling (MDS) analysis and heatmap clustering based on randomly selected differential expression genes (DEGs) can be seen in Fig. 2a and Fig. 2b, respectively. The complete list of identified DEGs between samples can be seen in Table S2 (10.6084/m9.figshare.19091954). Furthermore, these data can be used for further functional genomic studies related to inflorescence development or other related studies in oil palm species.

## 2 Experimental Design, Materials and Methods

### 2.1 Plant material and treatment

The control and complete defoliation treatment were applied to a sample of oil palm trees (15 years old) planted at the Gunung Sejahtera Ibu Pertiwi Plantation, Kalimantan Tengah, Indonesia. The complete defoliation treatment was conducted as Durand-Gasselin et al. [3] described. The control and complete defoliation treatment can be seen in Fig. 1. The term “control” refers to the fact that no treatment is conducted to the individual. On the other hand, the complete defoliation treatment consisted of removing all the leaves by pruning and conserving only the center unopened one to avoid tree death. Later, both trees were felled 45 days after the treatment [4]. Before the trees were felled, each leaf frond was marked with a corresponding leaf number to make it easier to count and sample the inflorescence. Samples were taken from three separate phases of the growing inflorescence, reflecting three consequential stages of the oil palm inflorescence (Fig. 1). The three different phases of inflorescence development from which tissues were taken are: the un-emitted inflorescence at leaf axil number +5 (stage 3), the inflorescence at leaf axil number -20 (stage 2), and the inflorescence at leaf axil number -27 (stage 1). Samples collected for transcriptomic analysis were immediately frozen in liquid nitrogen and stored at -80°C until further used.

### 2.2 RNA extraction and sequencing

Total RNA was isolated from 100 mg of tissues using the RNeasy Plant Mini Kit (Qiagen Inc. Valencia, CA, USA) according to the manufacturer's protocol. The quality and quantity of extracted RNA were measured using NanoDrop spectrophotometer (Thermo Fisher Scientific) and Qubit fluorometer (Invitrogen), followed by visualization on 0.8% agarose gel. Complete sequence library preparation and transcriptome sequencing for the Illumina HiSeq 2000 protocols were conducted by Macrogen, Inc. (Seoul, Korea). The FASTQ file generation was performed by Illumina Pipeline (CASSAVA) software v1.8.2 (Illumina Inc.).

### 2.3 Data analysis

FastQC version 0.11.5 software was used for quality-checked the sequenced reads. Given the high quality of the sequenced reads, we omitted the trimming procedure to prevent any potential biases, as previously reported [5]. The program HISAT2 (v2.1.0) [6] was used to align sequencing reads to the reference genome of oil palm (GCF_000442705.1). The gene abundances were quantified using StringTie (v1.3.4) [7]. The estimated read counts and calculated Fragments Per Kilobase Million (FPKM) were used for differential expression analysis. Statistical analysis of differential gene expression was conducted with DESeq2 (v1.18.1) [8]. Transcripts with a minimum two-fold change (FC) value and with a significance value of 0.01 after application of Benjamini–Hochberg false discovery rate (FDR) were considered as differentially expressed genes between control and defoliation treatment groups. All the samples were clustered using both multidimensional scaling (MDS) and heatmap analysis based on the relative expression of DEGs to investigate the overall expression patterns between control and defoliation treatment groups.

## Ethics Statements

Not applicable.

## CRediT Author Statement

**Ardha Apriyanto**: Conceptualization, Methodology, Formal analysis, Investigation, Resources, Data curation, Visualization, Writing, Reviewing, and Editing; **Walter Ajambang**: Conceptualization, Methodology, Investigation, Reviewing, and Editing.

## Declaration of Competing Interest

The authors declare that they have no known competing financial interests or personal relationships that could have appeared to influence the work reported in this paper.
